# Rare case of inguinal ureteral hernia in a child diagnosed by drip infusion pyelography-computed tomography

**DOI:** 10.1016/j.ijscr.2022.107007

**Published:** 2022-04-09

**Authors:** Toshifumi Hosoda, Kohei Hijikata, Shigeki Ishioka

**Affiliations:** Department of Surgery, Teikyo University Hospital, 2-11-1, Kaga, Itabashi-ku, Tokyo 173-8605, Japan

**Keywords:** Inguinal hernia, Sliding hernia, Ureteral hernia

## Abstract

**Introduction and importance:**

Inguinal hernias are the most commonly experienced disease in pediatric surgery. However, it is rare for the organs of the urinary system to prolapse as the contents of the hernia.

**Case presentation:**

We report a case of a 14-year-old boy with congenital paraperitoneal inguinal herniation of the ureter. Intraoperatively, we found an unfamiliar tubular loop structure arising from the deep inguinal ring in the left inguinal canal. The tubular structure, which may have been part of the ureter, was left in the inguinal canal to avoid damage. Postoperative drip infusion pyelography-computed tomography showed anatomical irregularity of the ureter in the inguinal canal. Follow-up in the 5th postoperative year showed no recurrence of hydrocele and complications associated with ureteral obstruction.

**Clinical discussion:**

Inguinal ureteral hernias are rarely reported in children. Paraperitoneal inguinal hernias are reported to be associated with vesicoureteral reflux and posterior urethral valve. Patients rarely present with symptoms like those observed in our case report. Whilst general surgical treatment is to return the ureter to the retroperitoneal space, we opted to leave the ureter in the inguinal canal to avoid unnecessary damage. However, this intraoperative management resulted in slight hematuria. The ureter should be placed back where it belongs, and postoperative monitoring using computed tomography may be important.

**Conclusion:**

This case provides valuable insight into preoperative diagnostic difficulties and intra- and postoperative management of an inguinal ureteral hernia in children, highlighting the importance of accurate diagnosis and appropriate surgical intervention in the treatment of this disease.

## Highlights

• Inguinal ureteral hernias are rarely reported in children.
• Preoperative diagnosis of inguinal ureteral hernias is difficult.
• An inguinal ureteral hernia may result in intraoperative technical difficulties.
• The possibility of an inguinal ureteral hernia should be considered under surgery.

## 1 Introduction

Inguinal hernias are the most commonly experienced disease in pediatric surgery. However, ureter prolapse as the contents of the hernia is significantly rare, and few pediatric cases of congenital inguinal herniation of the ureter have been reported [1]. Most cases with inguinal ureteral hernias are reported to occur in adults. We report a case of an inguinal hernia with ureteral prolapse in children.

## 2 Case report

A 14-year-old boy, who presented with left scrotal bulging, was referred to our hospital. He had a past medical history of left inguinal hernia surgery when he was one. According to the initial operation record, two vasa deferentia were confirmed, and one of them was significantly thick at 4 mm. The general operation for inguinal hernia could not be performed, and high ligation of the hernia sac could be performed at long last after detaching a severe adhesion of “some” spermatic cords. There were thus far no complications after the inguinal hernia surgery. During the initial physical examination, the patient had an egg-sized, smooth, and painless translucent solid mass in the left scrotum. No tumor was palpable in the groin, and both the silk glove sign and pump tests were negative. Ultrasonography revealed a large hydrocele (10 cm) around the left testis.

**Fig. 1:**
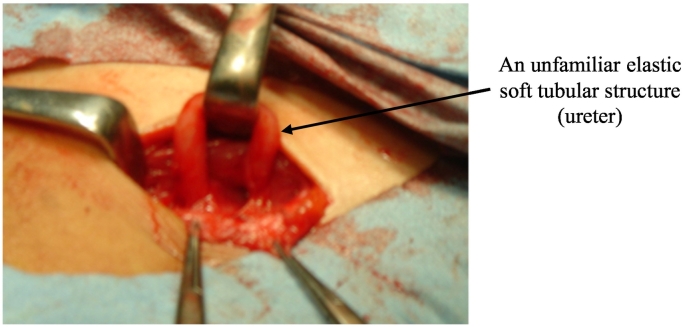
Intraoperative findings.

The procedure was performed under general anesthesia. A skin incision was performed along the previous surgical wound, and the inguinal canal was opened. The intestinal tract and a hernia sac were not shown in the inguinal canal. After detaching a severe adhesion of the spermatic cord, we detected an unfamiliar elastic soft tubular structure (thickness 4 mm; length 5 cm) which emanated from the internal inguinal ring separately from the spermatic cord and returned to the internal inguinal ring (Fig. 1). We opted only to incise and open the hydrocele in the scrotum; as we reasoned that the hydrocele showed no patency in the inguinal canal and was diagnosed as a noncommunicating scrotal hydrocele. Judging from its shape, the unusual tubular structure was most likely to be a ureter, but it was unrecognizable at that time. We adopted a policy to leave the tubular structure in the inguinal canal to avoid needless damage due to the detachment procedure and did not close the defect considering the risk of some obstruction in the inguinal canal.

**Fig. 2:**
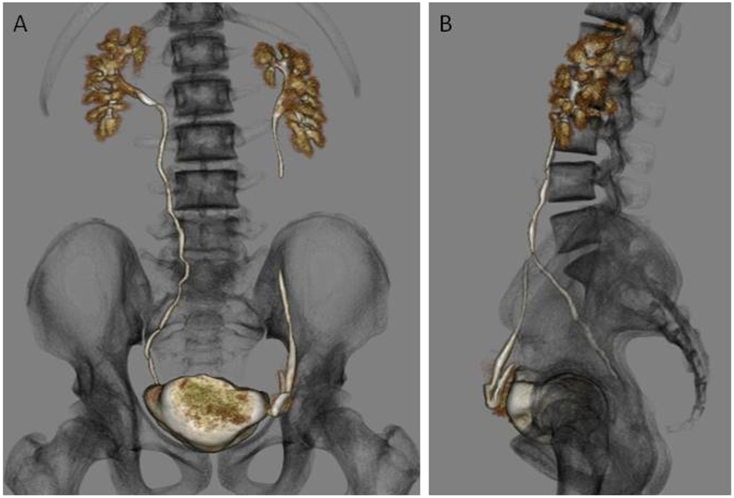
Postoperative drip infusion pyelography-computed tomography (DIP-CT). The left ureter reached the inguinal canal via the internal inguinal ring, turned over with a loop, and was connected to the bladder via the internal inguinal ring.

Postoperative drip infusion pyelography-computed tomography (DIP-CT) was performed to confirm the urinary system abnormalities. DIP-CT showed no abnormalities in the shape and position of both kidneys and no hydronephrosis. Nevertheless, the left ureter reached the inguinal canal via the internal inguinal ring, looped around, changed direction, and then connected to the bladder (Fig. 2). Based on the above DIP-CT findings, the patient was diagnosed with an inguinal ureteral hernia. He presented with slight hematuria immediately after the operation, but his urine gradually returned to normal. The patient was discharged on postoperative day one, without renal dysfunction. Follow-up in the 5th postoperative year showed no recurrence of hydrocele and complications associated with ureteral obstruction. This work has been reported in line with the SCARE 2020 criteria [2].

## 3 Discussion

**Fig. 3:**
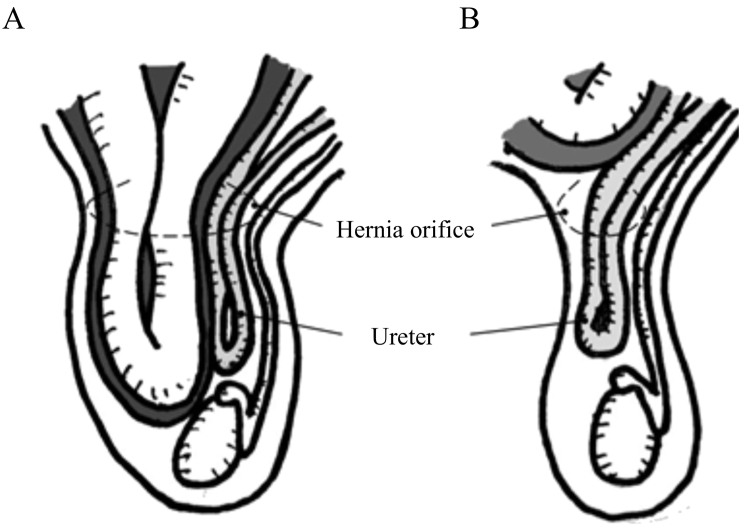
Types of inguinal ureteral hernias. Inguinal ureteral hernias are classified into two types: paraperitoneal (A) and extraperitoneal (B).

Since the first case of an inguinal ureteral hernia was reported by Leroux et al. in 1880, more than 140 cases of inguinal ureteral hernia have been thus far reported [3], [4], [5]. Most inguinal ureteral hernias are indirect hernias and are classified into two types based on their anatomical structure: the paraperitoneal type with a hernia sac and the extraperitoneal type without a hernia sac (Fig. 3) [6]. As shown in Table 1, the paraperitoneal type accounts for 80% of all cases and is acquired by males in their 40s to 60s. In paraperitoneal hernias, prolapse of the bladder and intestine are reported to be associated with the hernia sac. Conversely, in the extraperitoneal type, with retroperitoneal fatty tissue and no hernial sac, only the ureter is reported to prolapse [6]. The congenital, extraperitoneal type is thought to be related to abnormal development of the urinary system, such as abnormal development of the Wolffian duct and ureter, and adhesion of the gubernaculum testis to the ureter. Indeed, many cases of extraperitoneal hernias are associated with malformations of the kidney and urinary tract, such as wandering kidney and crossed renal ectopia [7]. In both types, there are often no specific symptoms other than distention of the inguinal region due to slipping of the ureter. However, extraperitoneal hernias may present with symptoms such as back pain due to obstruction or strangulation of the ureter or incarceration of the hernia [8]. In a study of preoperative diagnosis of inguinal ureteral hernias, one report demonstrated that 1 in 139 patients with inguino-scrotal hernias underwent preoperative ultrasonography to confirm ureteral dilatation [9]. Thus, preoperative diagnosis may be difficult; however, if signs of ureteral obstruction, such as hydronephrosis or hydroureter, are observed in cases of inguinal herniation, the possibility of an inguinal ureteral hernia should be considered.

**Table 1:** Clinical features of inguinal ureteral hernia.

Classification	Paraperitoneal type	Extraperitoneal type
Frequency	80%	20%
Etiology	Adhesion of the ureter to the peritoneum	Abnormal development of the ureter
Peak age of onset	40–60s	−
Hernia sac	+	−
Prolapse of another organ	+	−
Urological complication or anomaly	Rare	46%

Whilst most cases of inguinal ureteral herniation have been reported in adults, a few cases have been reported in children, as shown in Table 2 [10], [11], [12], [13], [14], [15]. Generally, the paraperitoneal type in adults is associated with rare urological complications or anomalies (Table 1), whereas the paraperitoneal type in children is more commonly associated with vesicoureteral reflux, posterior urethral valve, giant ureter, and polycystic dysplastic kidney (Table 2). Indeed, according to his operation record, a radical operation for a left inguinal hernia, hernia sac, and two vasa deferentia was confirmed in the inguinal canal, in which high ligation of the hernia sac was performed. Based on the fact that the ureter deviated from the natural anatomical position as shown by DIP-CT and the presence of the hernia sac had been confirmed, we diagnosed the patient with a congenital paraperitoneal ureteral sliding hernia. However, further investigations into urological complications and anomalies between congenital and acquired paraperitoneal types are required to elucidate the involvement of congenital factors.

**Table 2:** Reports of cases of inguinal ureteral hernia in children.

Author	Year	Age Gender Side	Type	Urological complication	Treatment
Bosschieter	2018	3 months Male Right	Paraperitoneal	Hydronephrosis megaureter vesicoureteral reflux	Ureterocutaneostomy
Handu	2012	18 months Male Right	Paraperitoneal	Solitary kidney	Transureteroureterostomy
Sripathi [9]	2011	10 months Male Left	Paraperitoneal	Vesicoureteral reflux	Vesicoureteral anastomosis
Burgu [10]	2010	4 months Male Left	Paraperitoneal	Posterior urethral valve	Vesicoureteral anastomosis
Powell [11]	1985	4 weeks Male Left	Paraperitoneal	Megaureter	Reduction to the retroperitoneal space
Morris [12]	1977	6 weeks Male Bilateral	Paraperitoneal	Multicystic dysmorphic kidney	–

General surgical treatment for inguinal ureteral hernia involves returning the prolapsed ureter into the inguinal region of the retroperitoneal space [14]. If not, postoperative hydronephrosis may develop due to torsion and flexion of the ureter; finally, the ureter may rupture. One report demonstrated that the ureter could be identified during the operation by carefully observing its peristaltic movement and aspirating clear fluid through a puncture of its contents [5]. Unfortunately, in our patient, we opted to leave the ureter in the inguinal canal to avoid unnecessary damage due to the detachment procedure. As a result, slight postoperative hematuria developed. Therefore, the ureter should be placed back into the preperitoneal space, and postoperative monitoring for urinary tract obstruction is important. If hydronephrosis or hydroureter develops, immediate removal of the obstruction is required.

## 4 Conclusion

This case provides valuable insight into preoperative diagnostic difficulties, as well as the intra- and postoperative management of an inguinal ureteral hernia in children, highlighting the importance of accurate diagnosis and appropriate surgical intervention associated with this disease.

## Consent

Written informed consent was obtained from the patient's parents for publication of this case report and accompanying images. A copy of the written consent is available for review by the Editor-in-Chief of this journal on request.

## Ethical approval

Ethical approval was not required for this case report in our institution.

## Funding

No funding.

## CRediT authorship contribution statement

Dr. Toshifumi Hosoda — corresponding author; collecting the data, writing the article, reviewing patient notes, writing articles, analyzing images, and approving the final submission.

Dr. Kohei Hijikata — collecting the data, reviewing patient notes, writing articles, and approving the final submission.

Dr. Shigeki Ishioka — collecting the data, writing the article, writing articles, and approving the final submission.

## Registration of research studies

N/A (this case is not a clinical trial).

## Guarantor

Dr. Toshifumi Hosoda.

## Declaration of competing interest

The authors declare no financial, personal, or other conflicts of interest that could induce bias.
